# Spontaneous cholecystocutaneous fistula secondary to xanthogranulomatous cholecystitis: a case report

**DOI:** 10.1186/s13256-022-03689-w

**Published:** 2022-12-15

**Authors:** Abdullah A. Almayouf, Hassan M. Ahmed, Adel A. Alzahrani, Abdulrahman H. Alashkar

**Affiliations:** 1 Department of Surgery, King Saud Hospital, Unayzah, Qassim Saudi Arabia; 2 Department of Surgery, Doctor Sulaiman Al-Habib Medical Group, Buraidah, Qassim Saudi Arabia

**Keywords:** Xanthogranulomatous cholecystitis, Laparoscopy, Cholecystocutaneous fistula, Case report

## Abstract

**Background:**

Xanthogranulomatous cholecystitis, a rare variant of cholecystitis, may infrequently be complicated by spontaneous cholecystocutaneous fistula.

**Case presentation:**

We report the case of a 75-year-old Saudi Arabian man who presented with “a painful area of redness” (cellulitis) over his right upper abdomen. Abdominal computed tomography revealed multiple collections, which were drained surgically. A discharging sinus was identified, and a fistulogram revealed cholecystocutaneous fistula during his follow-up visit. The patient underwent laparoscopic management and recovered uneventfully. Final histopathological evaluation confirmed acute-on-chronic xanthogranulomatous cholecystitis .

**Conclusions:**

Although rare, surgeons should consider cholecystocutaneous fistula in the differential diagnosis of anterior abdominal wall abscesses, particularly in patients with concurrent or background symptoms of gallbladder disease. We report the first case of laparoscopic management for cholecystocutaneous fistula in Saudi Arabia.

## Background

Biliary fistulization refers to the formation of an abnormal tract between the gallbladder and adjacent epithelial surfaces, which could be the gastrointestinal tract (internal fistula) or the abdominal wall (external fistula). External fistulization maybe spontaneous, post-traumatic, or iatrogenic in origin [1].

Spontaneous cholecystocutaneous fistula (CCF) is a rare complication of neglected gallbladder disease and is infrequent in modern surgical practice because symptoms associated with the gallbladder are unlikely to be ignored by patients or missed by physicians. This entity was first described by Thilesus in 1670, and to date, less than 100 cases have been reported in the literature [2]. We report a case of spontaneous CCF in an elderly man who was successfully treated laparoscopically.

## Case presentation

A 75-year-old Saudi Arabian man with a known clinical history of diabetes and hypertension presented to the emergency department with a 4-day history of “a painful area of redness” over his right upper abdomen. He denied any other symptoms at the time of presentation. The patient’s past history was remarkable for multiple episodes of colicky, transient, but tolerable upper abdominal pain associated with heavy meals. His past surgical history included bilateral inguinal hernia and hydrocele repair. He denied any relevant drug or family history.

Upon examination, the patient was ambulatory, conscious, and oriented. He looked well, not pale or jaundiced. He was afebrile with stable vital signs. Except for the area of redness over the right upper quadrant, his abdomen was soft, lax, nontender, and nondistended. The right upper abdomen showed a raised area of cellulitis (erythematous, warm to touch, and tender). A review of other systems revealed no significant findings.

Routine laboratory workup showed a slightly elevated white blood cell count of 10.4 × 10^9^/L (4–10 × 10^9^/L), and hypokalemia of 3.1 mmol/L (3.5–5 mmol/L). Liver function test results were within the reference ranges.

**Fig. 1:**
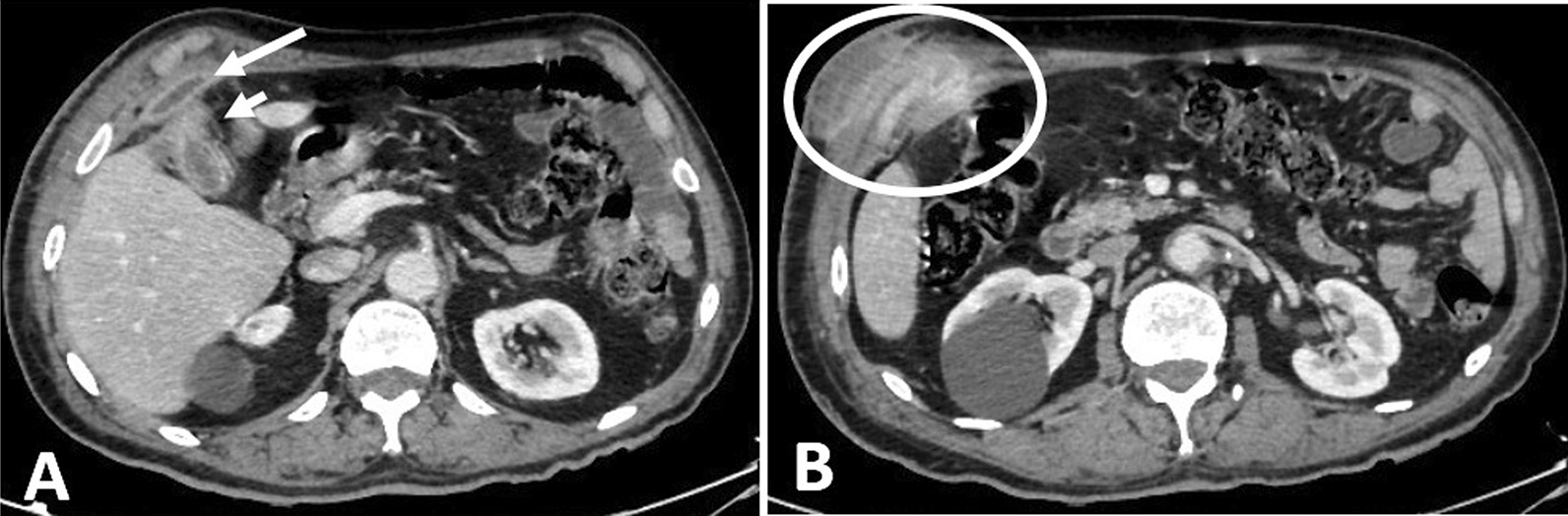
Computed tomography scan of the patient showing the superficial and deep collections at the right upper quadrant. **A** Illustrates the deep collection (long arrow) with multiple fat stranding surrounding the gallbladder (short arrow); notice the proximity between the collection and gallbladder with its inflammatory changes. **B** Illustrates the superficial collection with the fistulous tract (circle)

Abdominal computed tomography (CT) revealed multiple collections involving the anterior right upper abdominal wall with superficial and deep components between the abdominal wall muscles. The superficial component measured approximately 2.0 × 5.0 × 5.0 cm (anteroposterior, transverse, and craniocaudal dimensions), and the deep component measured approximately 1.5 × 5.0 × 8.5 cm (Fig. 1). Additionally, an inflamed gallbladder was observed in close proximity to the abdominal wall. We selected a two-staged surgical approach in this patient, with initial surgical drainage of the abscess and antibiotic therapy followed by interval cholecystectomy.

We performed incision and drainage of the abscess under local anesthesia. We drained about 20 mL of purulent content and sent a sample for culture and sensitivity testing, which later came back to be negative (that is, no growth). The patient’s postoperative course was uneventful, and he was discharged on oral antibiotics. He was pain free and had recovered uneventfully at the first postoperative visit at 2 weeks. However, examination showed an opening at the edge of the wound site with mucoserous discharge. Therefore, the patient was admitted for further evaluation.

**Fig. 2:**
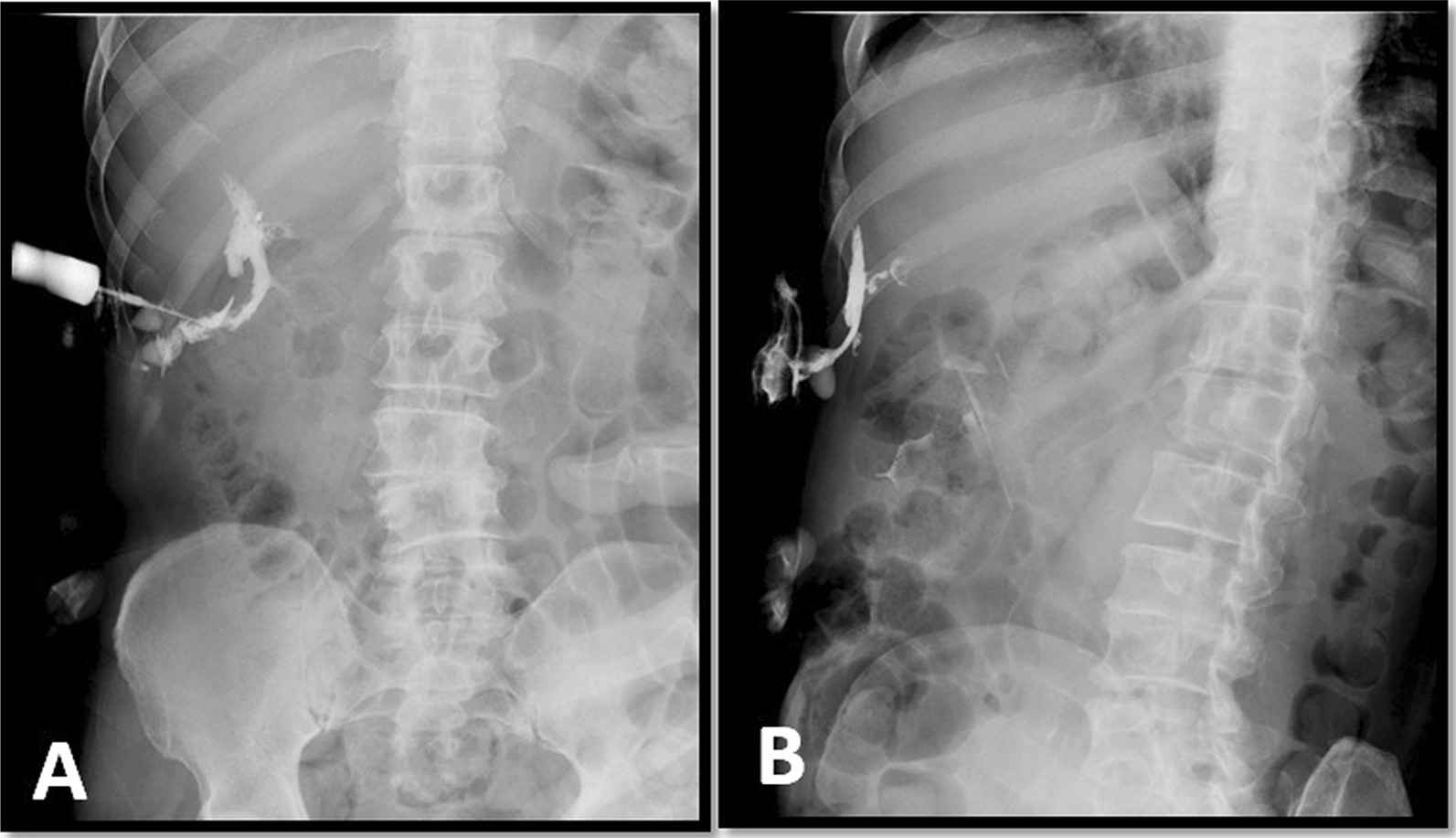
Fistulogram under fluoroscopy. **A** Demonstrates an injection of the contrast while the patient is supine and shows the sinus tract. **B** Demonstrates left lateral view and shows the C-shaped structure (gallbladder)

**Fig. 3:**
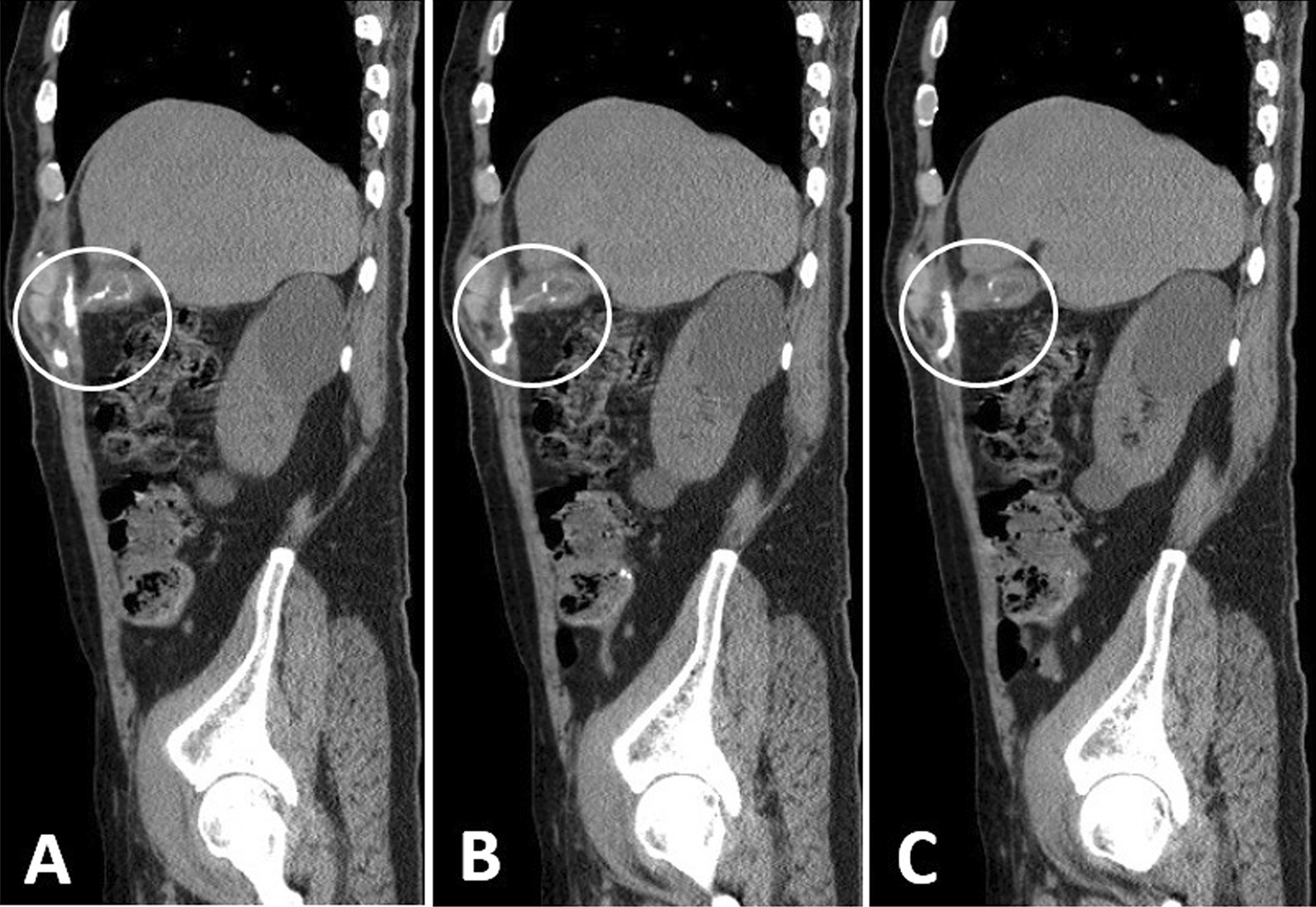
Sagittal view showing the presence of cholecystocutaneous fistula. **A**–**C** shows the fistulous track and its emergence into the gall bladder fundus (circles)

We performed fluoroscopy guided fistulography (Fig. 2), and observed free passage of the contrast agent into the subcutaneous tissue extending up and down, and a lateral view showed a sinus tract located medially. Plain abdominal CT performed in the same setting (Fig. 3) revealed accumulation of contrast material in the subcutaneous tissue of the right upper quadrant and a sinus tract filled with contrast and extension to the deep subcutaneous tissue. The contrast material was observed to pass through a small sinus tract and reached the gallbladder fundus and emptied into its lumen. The fundus was attached to the abdominal wall without a cleavage line. The gallbladder was contracted, without accompanying biliary dilatation. We diagnosed the patient with chronic cholecystitis and a communicating CCF and performed laparoscopic cholecystectomy and fistulous tract excision.

**Fig. 4:**
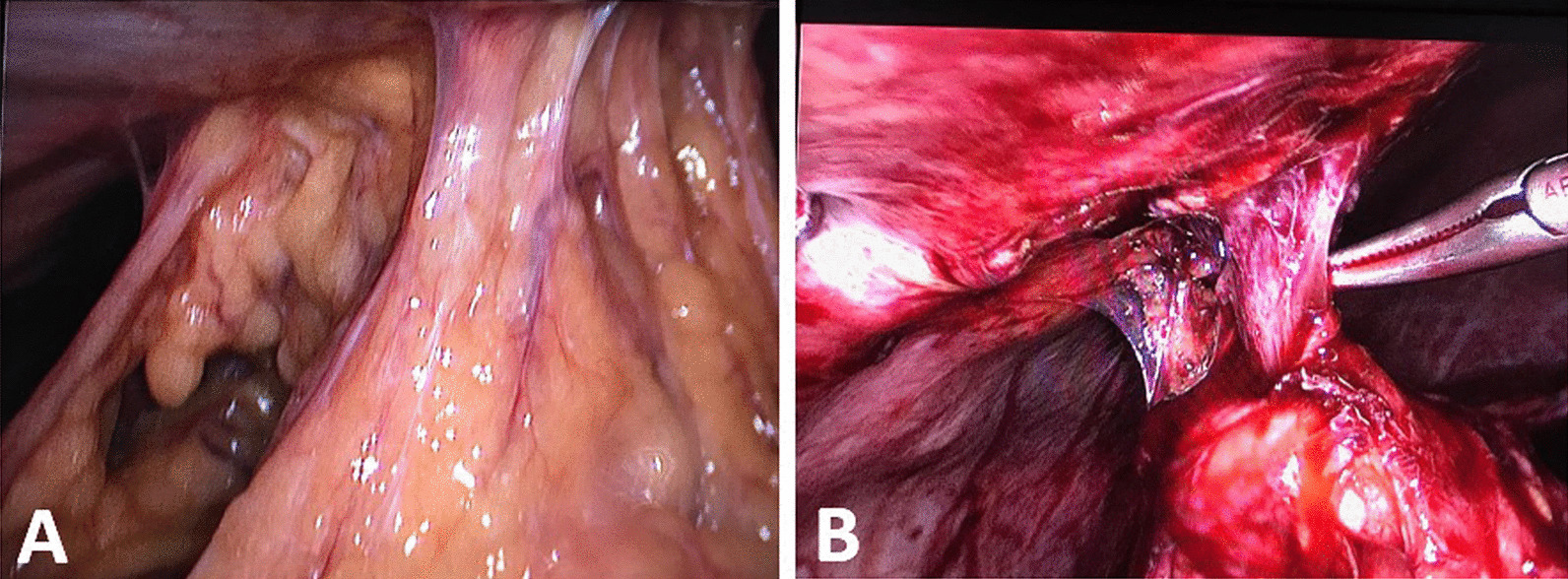
An illustration of intraoperative findings. **A** Describes the adhesions at the site of fistulous tract released by cautery and blunt dissection. **B** Shows the fistulous tract after release of the surrounding adhesions, which was thereafter divided

Intraoperatively, we performed meticulous dissection to separate adhesions around the fistulous tract until the fistulous tract was clearly visible (Fig. 4). The tract was divided near the abdominal wall, Calot’s triangle was exposed, and we placed a drain after successful cholecystectomy. We made an elliptical incision around the site of the external opening, excised the fistulous tract, and performed primary wound closure.

The patient showed an uneventful postoperative recovery. The drain was removed on the second postoperative day, and the patient was discharged. He was followed up 2 and 4 weeks postoperatively and recovered well with complete symptom resolution. Final histopathological evaluation confirmed diagnosis of acute-on-chronic xanthogranulomatous cholecystitis.

**Table:** 

Case timeline	
Visit 1 (time: 0)	Presented to the emergency department with abdominal wall cellulitis. Computed tomography scan showed abdominal wall collection and cholecystitis. Treated with abscess incision and drainage, and oral antibiotics. Planned for interval cholecystectomy
Visit 2 (week 2)	Discharging sinus noted. Fistulography demonstrated cholecystocutaneous fistula. Treated with laparoscopic cholecystectomy and fistulous tract excision. Discharged on postoperative day 2
Visit 3 (week 4)	Achieved full recovery. Histopathological diagnosis: of acute-on-chronic xanthomatous cholecystitis

## Discussion and conclusions

We present a case of spontaneous CCF, a rare complication of gallbladder disease. Overall, fewer than 100 cases have been reported in the literature [2]. To the best of our knowledge, only one similar case has been reported in Saudi Arabia. Our patient is the first to be treated laparoscopically [3].

Most cases of spontaneous CCF are associated with neglected calculous gallbladder disease, as observed in our patient. However, a small number of studies have reported that such complications were secondary to gallbladder adenocarcinoma [4, 5]. Similar to the gender-based predilection for gallstone disease, spontaneous CCF is more commonly seen in female patients. Unlike the usual predominance of gallstones in young women, CCF is more common in elderly women (although it may occur in men), which reflects patients’ longstanding “neglect” of their symptoms, which is typically associated with the development of this complication. In addition to age and neglected gallbladder disease, other factors that might contribute to the development of CCF include a history of steroid use and immunocompromised states.

The clinical manifestations vary and depend on the patient’s status and time of presentation; however, most patients present with systemic (fever, nausea, and/or vomiting) and local (pain, swelling, and/or discharge) features [6]. Although our patient had a history of gallbladder colic, he primarily sought consultation for evaluation of abdominal wall redness. Additional, more specific features might include stones or bile draining through the external opening of the fistula, which was not noted in this case.

Imaging is necessary for diagnostic confirmation. Ultrasonography may show abscesses, gallstones, and evidence of inflammation. However, it is unlikely to demonstrate a fistulous tract, and CT (or magnetic resonance imaging) is necessary. Additionally, a fistulogram (radiography or CT) may be a useful aid in confirming and accurately assessing the fistulous tract. Magnetic resonance cholangiopancreatography is an essential diagnostic modality if biliary obstruction is suspected. Biliary obstruction should be suspected on the basis of clinical findings (jaundice) or investigation results (liver function test abnormalities or evidence of common bile duct dilatation).

Conclusive guidelines are unavailable for the diagnostic approach and optimal treatment of CCF. Therefore, an individualized management regimen should be implemented on the basis of the underlying etiology, time, and nature of presentation, and treatment largely depends on the surgeon’s expertise. Decision-making should necessarily be based on thorough discussion with the patient.

The management of the acute phase of this condition includes resuscitation, pain control, and antibiotic therapy. Watchful waiting to allow healing and spontaneous closure of the fistulous tract may be considered in patients deemed unfit for surgery. However, the outcomes of such approaches remain unclear. Several studies recommend an open approach, considering its feasibility and lower complication rates [7, 8]. We are in agreement with this strategy and concur that laparoscopic surgery performed by surgeons with the required expertise may be a feasible approach for effective management of CCF in select patients and offers the known advantages of laparoscopic surgery. Malik *et al.* have described laparoscopic surgery in such cases [9]. In addition, two more similar studies reported laparoscopic treatment for this condition [7, 10].

Finally, following the final histopathological diagnosis is of the utmost importance, as gallbladder carcinoma is a possibility. In our case, histopathological evaluation confirmed diagnosis of acute-on-chronic xanthogranulomatous cholecystitis (XGC). XGC is a rare inflammatory disease of the gallbladder. It is characterized by destructive inflammation and fibrosis of the gallbladder wall and sometimes adjacent structures. This makes it difficult to differentiate from carcinoma radiologically, hence the importance of histopathological evaluation. It presents similarly to calculous gallbladder disease and has comparable complications, yet they are more common [11].

This case report highlights that surgeons should consider CCF in the differential diagnosis of anterior abdominal wall abscesses, particularly in patients with concurrent or background symptoms of gallbladder disease. Being a rare differential, some features, such as history of longstanding and neglected gallbladder disease or the presence of bile drainage through the external opening, might further point to this diagnosis. Imaging is necessary for diagnostic confirmation, and fistulography is recommended. Decision-making regarding the optimal surgical approach for this rare complication warrants careful evaluation of the disease stage and the patient’s general health condition, and should be based on the surgeon’s expertise and the patient’s preference.

## Data Availability

All data generated are included in this published article.
